# Posttranslational Modification of Human Glyoxalase 1 Indicates Redox-Dependent Regulation

**DOI:** 10.1371/journal.pone.0010399

**Published:** 2010-04-29

**Authors:** Gerd Birkenmeier, Christin Stegemann, Ralf Hoffmann, Robert Günther, Klaus Huse, Claudia Birkemeyer

**Affiliations:** 1 Faculty of Medicine, Institute of Biochemistry, University of Leipzig, Leipzig, Germany; 2 Faculty of Chemistry and Mineralogy, Center for Biotechnology and Biomedicine, Institute of Bioanalytical Chemistry, University of Leipzig, Leipzig, Germany; 3 Faculty of Biosciences, Pharmacy and Psychology, Institute of Biochemistry, University of Leipzig, Leipzig, Germany; 4 Leibniz Institute for Age Research–Fritz Lipmann Institute e.V., Jena, Germany; 5 Faculty of Chemistry and Mineralogy, Institute of Analytical Chemistry, University of Leipzig, Leipzig, Germany; Deutsches Krebsforschungszentrum, Germany

## Abstract

**Background:**

Glyoxalase 1 (Glo1) and glyoxalase 2 (Glo2) are ubiquitously expressed cytosolic enzymes that catalyze the conversion of toxic α-oxo-aldehydes into the corresponding α-hydroxy acids using L-glutathione (GSH) as a cofactor. Human Glo1 exists in various isoforms; however, the nature of its modifications and their distinct functional assignment is mostly unknown.

**Methodology/Principal Findings:**

We characterized native Glo1 purified from human erythrocytes by mass spectrometry. The enzyme was found to undergo four so far unidentified posttranslational modifications: (i) removal of the *N*-terminal methionine 1, (ii) *N*-terminal acetylation at alanine 2, (iii) a vicinal disulfide bridge between cysteine residues 19 and 20, and (iv) a mixed disulfide with glutathione on cysteine 139. Glutathionylation of Glo1 was confirmed by immunological methods. Both, *N*-acetylation and the oxidation state of Cys^19/20^, did not impact enzyme activity. In contrast, glutathionylation strongly inhibited Glo1 activity *in vitro*. The discussed mechanism for enzyme inhibition by glutathionylation was validated by molecular dynamics simulation.

**Conclusion/Significance:**

It is shown for the first time that Glo1 activity directly can be regulated by an oxidative posttranslational modification that was found in the native enzyme, i.e., glutathionylation. Inhibition of Glo1 by chemical reaction with its co-factor and the role of its intramolecular disulfides are expected to be important factors within the context of redox-dependent regulation of glucose metabolism in cells.

## Introduction

Glyoxalases (Glo1, E.C. 4.4.1.5, and Glo2, E.C.3.1.2.6) constitute an ubiquitous detoxification system that protects against cellular damage caused by reactive 2-oxo-aldehydes such as methylglyoxal (MGO). MGO originates mainly from non-enzymatic degradation of triose phosphates but is also formed during amino acid metabolism and acetone oxidation [1]. Glo1 (*S*-D-lactoylglutathione lyase) and Glo2 (hydroxyacyl glutathione hydrolase) convert the spontaneously formed hemithioacetal between glutathione (GSH) and MGO into D-lactate and free GSH. Thus, GSH acts as a physiological cofactor of Glo1 [2]. Glo1 is known to play a role in many diseases including diabetes mellitus [3], Alzheimer's disease [4], and cancer [5].

Although Glo1 is ubiquitously expressed, limited information is available on how this enzyme is regulated in human cells. Particularly, cells with increased glucose metabolism are suggested to protect themselves against cellular damage by MGO through up-regulation of Glo1 activity. That is most tumor cells, such as cells derived from prostate, breast, and colon cancer, displayed increased expression of Glo1 in conjunction with enhanced anaerobic breakdown of glucose to lactate [5]. However, in expression studies, cellular effects expected from reduced Glo1 activity and Glo1 knockdown such as increased MGO concentration revealed rather discordant findings [6], [7]. Within this context, GSH was proposed to exhibit other functions beyond its mere anti-oxidative properties as enzyme co-factor and redox-regulator [8].

The human enzyme Glo1 is a homodimeric Zn^2+^- dependent isomerase with two identical active sites at its dimer interface; the two monomers are associated by non-covalent bonds [9]. The derived translation product of the human GLO1 gene (Q04760) consists of 184 amino acids. There are two genetic variants of Glo1 differing at position 111 with a glutamate-alanine substitution (E111A) resulting in subsequent formation of three Glo1 isozymes in heterozygotes. After putative posttranslational removal of *N*-terminal methionine as suggested by Ridderstrom [10], the calculated average masses of the monomers are 20,647.4 Da and 20,589.4 Da (E111A) respectively, deduced from nucleotide sequences. The molecular mass of the dimer was 46 kDa determined by gel permeation chromatography [11].

Beside the known genetic variants (allozymes), a couple of different isoforms of Glo1 have been described [12], but only few studies addressed the nature of the enzyme's polymorphism. The *N*-terminal alanine residue was found to be blocked by an unknown modification in the mature human protein [10]. Further, at least four putative phosphorylation sites were deduced from the amino acid sequence [11]. Indeed, phosphorylated Glo1 has been recently detected in fibrosarcoma cell line L929 upon treatment by tumor necrosis factor α (TNFα) [12], [13]. A distinct phosphorylation site was identified at Thr^106^ [14]. While phosphorylation seemed to impact the pattern of MGO-derived advanced glycation end products (AGE), no effect upon the catalytic activity of the enzyme itself was found that would explain the elevated MGO concentration found in TNFα-treated cells [13].

Reversible inactivation of Glo1 was observed upon exposure of cells to extra-cellular nitric oxide (NO) which was strictly dependent on the level of GSH indicating that Glo1 is an NO-responsive protein [15], [16]. It was suggested that the observed inhibiting modification of the enzyme was brought about by nitrosylation of distinct amino acid residues near the active site of Glo1. Studies on Glo1 Cys-mutants revealed that cysteine residues, primarily Cys^139^, may be involved in the NO-responsiveness of Glo1 [12].

To our knowledge, other covalent modifications of the human enzyme have not yet been described. Most investigations on Glo1 did not rely on mass spectrometry and its unique potential to identify posttranslational modifications by interpretation of characteristic mass differences and their localization after digest of the protein. There are only few reports on Glo1 applying mass spectrometry at all, such as studies of Glo1 from *E. coli* which *inter alia* described the non-covalent dimerization of the enzyme as a function of pH [17], [18].

In the present study, we identified and localized four posttranslational modifications of Glo1 by means of mass spectrometry and immunological approaches: (i) removal of *N*-terminal methionine, (ii) *N*-terminal acetylation, (iii) a vicinal disulfide bridge at Cys^19/20^, and (iv) glutathionylation at Cys^139^. Glutathionylation of Glo1 strongly inhibited enzyme activity at physiologically relevant concentration of GSH. Our results suggest direct regulation of the enzyme in response to the cellular redox state, which will have crucial importance for elucidating the functions of Glo1 *in vivo*.

## Results

### Evidence of posttranslational modification of Glo1 by ESI-FTICR-MS analysis

**Figure 1:**
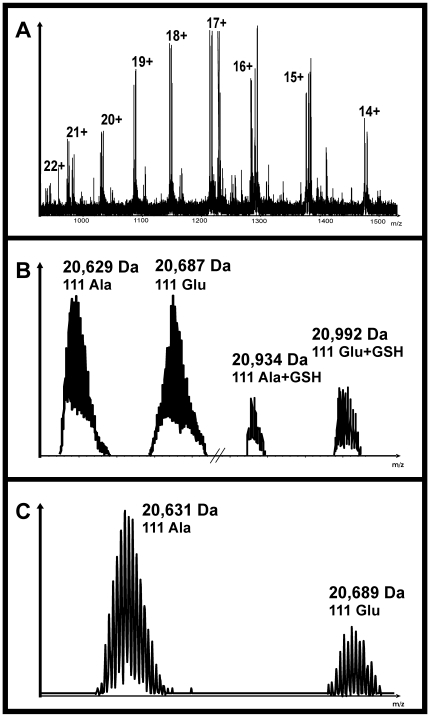
Glo1 ESI-FTICR mass spectrum. (A) Original spectrum, peaks are labeled according to their charge. The two isoforms of Glo1 and the corresponding glutathionylated forms are clearly separated at a resolution of >25,000. (B) Deconvoluted spectrum of Glo1, m/z axis not to scale. Peaks are labeled with the average mass. Processing by XMass Software clearly extracts the two separated allozymes. Right-hand side, the corresponding glutathionylated forms are shown. (C) Deconvoluted spectrum of a purified Glo1 preparation after β-mercaptoethanol treatment. The determined average mass differs by 2 Da compared with the native monomeric forms, the glutathionylated forms were not detected in this preparation.

Purified Glo1 from human erythrocytes was subjected to analysis by direct-infusion ESI-FTICR (Figure 1A). At acidic conditions, a charge distribution of +14 - +22 was observed. ESI mass deconvolution demonstrates the presence of two species with an average molecular mass of 20,629.7 Da and 20,687.4 Da (Figure 1B), respectively, exhibiting a mass difference of 58 Da. This mass difference would be in accordance with the two possible monomers of the enzyme that differ at position 111, carrying either alanine or glutamic acid. To our knowledge, this is the first time the exact molecular mass of the two allelic monomers of human Glo1 was determined by mass spectrometry.

In fact, after suggested posttranslational methionine removal [10], average masses of the two monomer allozymes were supposed to be 20,589.3 Da or, 20,647.4 Da, respectively, deduced from the nucleotide sequence of human Glo1 [19]. Compared with the theoretical value, we found a mass difference of +40 Da proposing posttranslational modification of the enzyme. Along with the two peaks of the monomer allozymes in the deconvoluted spectrum, we observed two additional peaks of 1/5 signal intensity with a mass difference of +305 Da compared to each of the two monomers (Figure 1B). The signal intensities of these “twin” peaks were different from preparation to preparation, ranging from not detectable till up to three times higher than the non-modified form.

After β-mercaptoethanol (β-ME) treatment, subsequent analysis by direct-infusion ESI-FTICR mass spectrometry followed by ESI mass deconvolution obtained average mass values of 20,631.8 Da and 20,689.5 Da for the monomers of the two allozymes (Figure 1C) resulting in a net-mass difference of 42 Da instead of the expected 40 Da found earlier. The mass difference of 2 Da after reduction was attributed to the presence of an intramolecular disulfide in a first instance. The M+305-Da peaks were not detected which indicated an oxidative modification of the enzyme.

### Posttranslational modifications of Glo1 were localized by MALDI-TOF/TOF analysis and nano-LC-ESI-MS/MS

**Figure 2:**
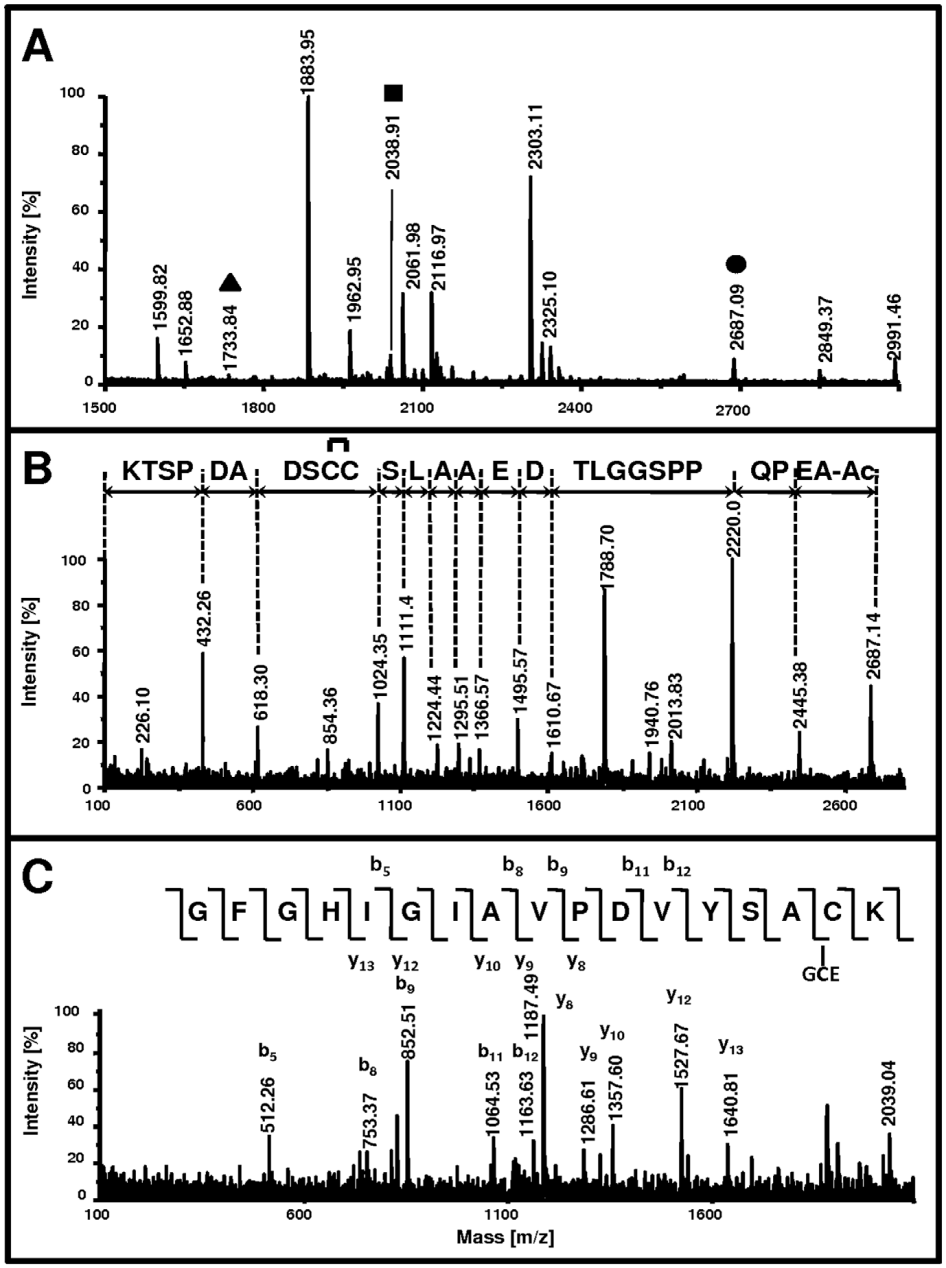
MALDI-TOF MS analyses of the non-reduced tryptic digest of Glo1. (A) Full Scan, m/z range 1500–3000. The presented m/z range of the MALDI-TOF spectrum contains the three important peptide fragments to this investigation. The first ion, [M+H]^+^ = 2687.09 (•), represents the *N*-terminus with *N*-acetylation and a vicinal disulfide-bridge on Cys^19/20^. The second peptide, [M+H]^+^ = 2038.91 (▪), contains the suggested binding site for GSH, namely Cys^139^. The third ion, [M+H]^+^ = 1733.84 (▴), is the same peptide, non-glutathionylated. (B): Tandem-MS analysis of (M+H)^+^ = 2687.09, the *N*-terminal peptide of Glo1, the sequence (HAc)-AEPQPPSGGLTDEAALSCCSDADPSTK-(OH) carrying the vicinal disulfide. D-E-A-A-S-L identified the peptide being the *N*-terminal tryptic cleavage product of Glo1. The y-series reveals a mass difference of 42 Da (acetylation) between the precursor ion and the last y-ion (y_27_) at the *N*-terminus. The 2 Da mass shift for the fragment C-C-S-D (y_6_ and y_10_), suggests a vicinal disulfide-bridge between the two cysteines. (C) Tandem-MS analysis of [M+H]^+^ = 2038.91, the tryptic peptide with glutathionylated Cys^139^. The y- and b-series confirm the identity of the peptide H-GFGHIGIAVPDVYSAC(GCE)K-(OH) with the partial sequence of V-DP-V-A-IG-I. Up to b_12_, the fragment pattern matches the non-modified series of the *N*-terminus of the peptide, but the mass difference of 874 instead of 569 between the b_12_ fragment and the precursor ion suggests a covalent attachment of GSH to this peptide sequence before GFGHIGIAVPDV. The y-series suggests GSH-attachment before y_8_, i.e. before GFGHIGIAV.

The peptide mass fingerprint (PMF) obtained with MALDI-TOF/TOF-MS/MS analysis of a tryptic digest of the non-reduced enzyme preparation (Figure 2) was identified as Glo1 with a score of 84 searched against SwissProt 54.7 database using an in-house mascot search engine [20].

In agreement with ESI-FTICR analysis of the native preparation, fragment m/z 2687.5 matched the expected mass for the *N*-terminal fragment increased by 40 Da (Figure 2A). As illustrated in Figure 2B, tandem mass spectrometry of the fragment m/z 2687.5 displayed the distinct y-series D-E-A-A-L-S which unambiguously confirmed the *N*-terminal tryptic peptide of Glo1, H-AEPQPPSGGLT*DEAALS*CCSDADPSTK-OH.

We found two discrepancies in the specific mass differences between the MS/MS-fragments of that peptide compared with the theoretical values deduced from sequence data. These were (i) confirmation of Met^1^ removal evident by comparison with the expected overall peptide mass of the *N*-terminus; (ii) the observed mass difference of 406 Da instead of 408 Da between m/z 1024 and 618 for the C-C-S-D-fragment of the *N*-terminus that suggested a disulfide-bridge located between these two vicinal cysteine residues; and (iii) the mass difference of 242 Da instead of 200 Da between m/z 2687 and 2445 as the theoretical deduced value for the *N*-terminal fragment A–E (Figure 2B), suggesting an acetylated terminal alanine.

*N*-terminal posttranslational modification of human Glo1 has been reported earlier by Ridderstroem et al. [10]. This *N*-terminal modification had no impact on enzymatic activity and was absent in the recombinant enzyme in *E. coli*, indicating a modification specific to eukaryotic cells. We identified this modification here being an *N*-terminal acetylation. In *E. coli*, *N*-terminal acetylation of proteins does not occur, while up to 85% of eukaryotic proteins are modified with an acetyl group after *N*-terminal cleavage of methionine [21].

We searched in the non-reduced preparation for other cleavage products that masses were increased by the observed mass difference of 305 Da and identified m/z 2038.9 as a potential candidate for the peptide H-GFGHIGIAVPDVYSACK-OH plus 305 Da (Figure 2A). H-GFGHIGIAVPDVYSACK-OH with an expected m/z 1734 was detected in the digest as well. Accurate mass measurement by direct infusion ESI-FTICR analysis confirmed the peak matching the expected mass for that peptide assuming a disulfide bond with GS(H) on Cys^139^, with m/z 510.48970 and a deviation of 0.4 ppm for the fourfold-charged and with m/z 680.31777 and a deviation of 1.3 ppm for the threefold-charged species, respectively.

The b- and y-fragments of MALDI-TOF/TOF-MS/MS of m/z 2038.9 were in accordance with this assumption (Figure 2C): the mass differences in the y-series with y_8_ to y_13_ and the b-series b_5_ to b_12_, i.e. the sequence corresponding to I-GI-A-V-PD-V, confirmed the peptide H-GFGH*IGIAVPD*VYSACK-OH +305 Da. All mass differences of y-ions to the precursor ion m/z 2038.9 were shifted by an increase of 305 Da from y_8_ on, i.e. y_13_ exhibited an m/z 1640.8 instead of the theoretical m/z 1336, while the b-series was in accordance with the theoretically expected values up to b_12_. This indicated a covalently attached 305 Da- modification near the C-terminus of the peptide involving the YSACK residue. After reduction and alkylation of the tryptic digest and analysis by MALDI-TOF/TOF-MS/MS, the target fragment at m/z 2038.9 was not detected; only the alkylated GFGHIGIAVPDVYSACK of m/z 1790 was found instead indicating the presence of a disulfide as modification of this peptide fragment.

**Figure 3:**
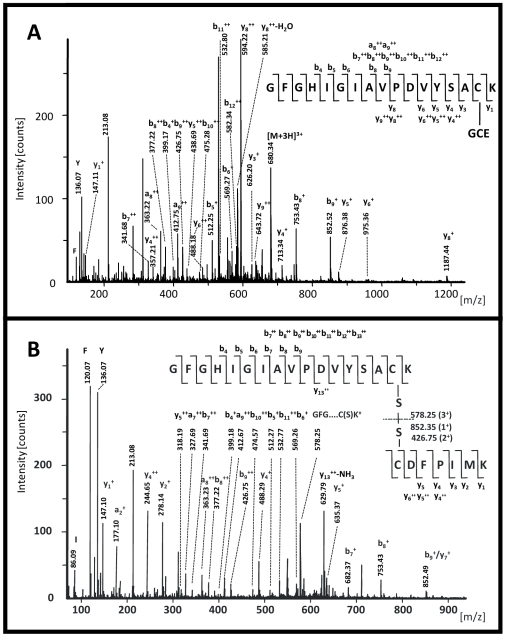
Nano-LC-ESI-qTOF-MS/MS analyses. (A) Tryptic peptide with glutathionylated Cys^139^ (m/z 2038.9) with corresponding b- and y-series. The y- and b-series confirm the identity of the peptide H-GFGHIGIAVPDVYSAC(GCE)K-(OH) with the partial sequences of I-G-I-A-V-P-D-V and PD-V-Y-S-AC(GCE)-K, respectively. Up to b_12_, the fragment pattern matches the series of the *N*-terminus of the peptide, the mass difference of 479 Da instead of 174 Da for the y_3_-y_1_ fragment locates the attachment of GSH to this peptide sequence at Cys^139^. (B) Disulfide bridge between the two tryptic peptides H-GFGHIGIAVPDVYSACK-(OH) and H-CDFPIMK-(OH) (Cys^61/139^, m/z 2584.3). The sequences of b_4_ up to b_13_ and y_1_ to y_6_ unambiguously identify the two peptides; the presence of y_13_ confirms the covalent disulfide bond.

These results were confirmed by nano-LC-ESI-QqTOF-MS/MS analysis (Figure 3A) where the b-series clearly confirmed the peptide sequence of I-G-I-A-V-P-D-V-Y and the corresponding y-series unambiguously restricted the site of a 305 Da- modification to the C-K sequence at the *C*-terminus.

Nano-LC-ESI-QqTOF-MS/MS analysis further revealed a fifth, optional modification of Glo1, namely another disulfide bridge between H-GFGHIGIAVPDVYSACK-OH and H-CDFPIMK-OH, i.e. Cys^139^ and Cys^61^ (Figure 3B). According to the b- and y-series obtained after MS/MS, these two tryptic peptides evidently assembled the observed species at m/z 2586. Thiol-disulfide exchange has already been shown to occur in several proteins during sample preparation [22], [23], in particular when thiol and disulfide are closely located to each other which is not the particular case with Cys^139^ and Cys^61^ [24]. Often, these artifacts are observed at much lower intensity than natively bound disulfides, which would be in accordance with the fact that we did not detect this tryptic fragment in our MALDI-TOF analyses or nano-LC analysis after alkylation only without reduction. However, disulfide exchange of Cys^139^ or Cys^61^ involving Cys^19^ or Cys^20^ was not observed.

### The vicinal disulfide bridge does not affect enzyme activity

Following treatment of reduced Glo1 with phenylarsine oxide (PAO) which is known to react specifically with vicinal sulfhydryl groups of proteins to form thioarsine rings [25], we found no change in enzyme activity (data not shown). These results indicate that modifying the vicinal disulfide alone is not sufficient to impact enzyme activity of Glo1.

### Glutathionylation was confirmed by immuno-detection of bound GSH

**Figure 4:**
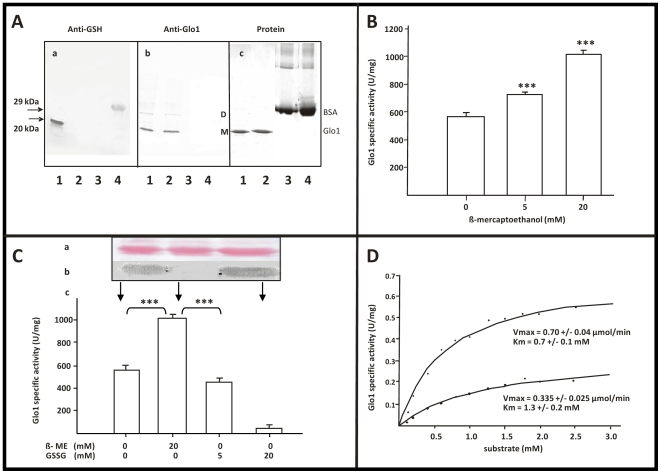
Glutathionylation of Glo1 by immunoreactivity and activity assays. (A) Anti-GSH mab display immunoreactivity against native Glo1. Purified human Glo1 separated by SDS-PAGE under non-reducing conditions was probed with the anti-GSH mab (a) or anti-Glo1 mab (b), respectively, followed by incubation with HRP-labelled goat anti-mouse Ig. Protein staining was accomplished by Coomassie Brilliant Blue R250 (c). (M) and (D) represent the monomer and dimer of Glo1. Lane 1: Glo1: 10 µg (a); 0.5 µg (b) and 5 µg (c); lane 2: Glo1: 10 µg +20 mM β-ME (a); 0.5 µg (b) and 5 µg (c); lane 3: BSA: 20 µg (a); 20 µg (b) and 20 µg (c); lane 4: BSA-GSH: 20 µg (a); 20 µg (b) and 20 µg (c). (B) Activation of Glo1 by β-ME. Glo1 was incubated with increasing concentrations of β-ME and activity was assayed after dialysis. Data is presented as the mean ± SD of three independent experiments. (*** = p<0.001; * = p<0.05 vs. blank). (C) Incubation of Glo1 with oxidized glutathione causes enzyme inhibition. Activity of reduced Glo1 was evaluated after incubation with 5 mM and 20 mM GSSG. The native, β-ME-treated (20 mM), and GSSG-treated (20 mM) enzyme, respectively, were subjected to non-reducing SDS-PAGE followed by Western blotting. The membrane was stained for protein with Ponceau S (a) and probed with the anti-GSH antibody (b). The enzyme activity of all samples was measured (c). Data are presented as mean ±SD of three independent experiments. (*** = p<0.001). (D) Kinetic analysis of native and reduced Glo1. Enzyme activity of 70 mU Glo1 was determined after incubation with increasing equimolar concentrations of MGO/GSH: non-treated enzyme (○);β-ME-treated enzyme (▪). Kinetic data are expressed as V_max_ (µmol/min) and Km (mM) values.

Further confirmation of glutathionylation of Glo1 was obtained by Western blotting with anti-GSH antibodies (Figure 4A). Anti-GSH antibodies were reactive to native Glo1 and BSA-GSH prepared as control, while no immuno-reactivity was observed when native Glo1 was incubated with β-ME prior to electrophoresis or BSA samples were probed with anti-Glo1 antibodies. These results clearly indicate that Glo1 is indeed a target protein for glutathionylation.

The observed glutathionylation at Cys^139^ is unlikely to be an artifact. Purification of the enzyme was carried out under non-denaturing, reducing conditions strictly avoiding any use of GSH. Furthermore, we found no evidence in our data that glutathionylation happens on any other cysteine of our protein. Human Glo1 has four cysteine residues, of which Cys^139^ is conserved from bacteria to human among the known species. Cys^19^ and Cys^20^ were found so far in mouse, rat, simian, and human Glo1, while Cys^61^ is unique to humans and possibly could be involved in substrate binding inside the hydrophobic pocket [24]. The vicinal Cys^19^ and Cys^20^ are probably not accessible due to their protecting disulfide bridge, but Cys^61^ would be generally amenable to bind GSH.

### Incubation of Glo1 with GSSG alters enzyme activity

We measured the activity of purified Glo1 upon incubation with a reducing agent (Figure 4B). Enzyme activity was increased up to 80% in the presence of β-ME in a concentration-dependent manner. We observed variation in specific activity and degree of β-ME-responsive activation in different enzyme preparations. To show the reversibility of activation by provoking thiol-disulfide exchange with the active enzyme species (denominated as Glo1-A), we incubated Glo1-A with increasing concentrations of GSSG and found almost complete inactivation of the enzyme (denominated as Glo1-B, Figure 4C). These results were corroborated also by Western blot. Moreover, reduction by β-ME was found to change the kinetic parameters of the enzyme preparation toward higher V_max_ and lower K_m_ values indicating that the substrate affinity strongly depends on the degree of glutathionylation (Figure 4D).

In conclusion of our results, we suggest that Glo1 exists *in vivo* in a non-glutathionylated, highly active form (A-enzyme), and a glutathionylated, less active, form (B-enzyme).

## Discussion

The cytoplasm is thought to be a reducing environment and, hence, disulfides are expected to be deliberately formed by oxidation involving specialized systems, such as the thioredoxin system, should they occur. Therefore, to find disulfide bonds and glutathionylation in Glo1 under regular conditions (assuming it is mostly cytoplasmic) clearly indicates a redox-regulation mechanism of enzyme activity.

### Glutathionylation regulates Glo1 activity

Since its discovery, glutathionylation of enzymes and transcription factors is being recognized as a central mechanism by which changes in the intracellular redox state may be transduced into functional cellular responses [26]–[28]; interaction of GSH with proteins was already suggested in 1985 by Grimm et al. [29].

Glutathionylation is mostly known to inactivate proteins, such as glyceraldehyde-3-phosphate dehydrogenase [30], human p53 [31], and NFκB [32], whereas activated few others such as human oncogene Ras [26], [28]. In particular, several glycolytic enzymes were found to be regulated by glutathionylation, such as glyceraldehyde-3-phosphate dehydrogenase, aldolase, phosphoglycerate kinase, pyruvate kinase, triose phosphate isomerase, and lactate dehydrogenase [33], [34]. It was further suggested that glutathionylation could coordinate cellular metabolism in response to oxidative stress by modulating glycolysis [34]. Interestingly, Fratelli et al. found an unidentified protein of 46 kDa also to be glutathionylated [35], i.e. the same molecular mass as the Glo1 dimer, and suggested that there are proteins “constitutively” glutathionylated in absence of oxidative stress. Moreover, the second enzyme of the glycolysis-associated Glo system, Glo2, has been found earlier to be glutathionylated *in vitro* but a physiological importance was deemed unlikely [36].

We demonstrate here that human Glo1 can be reversibly glutathionylated and its activity is suppressed by glutathionylation. Within cells, the ratio of GSH/GSSG is assumed to be about 100∶1 but in fact it may deviate from that considerably upon oxidative stress [28]. An inhibiting influence of excess GSH on Glo1 activity was reported earlier as competitive inhibition to the hemi-mercaptal substrate [37], while low concentration was found to favor MGO detoxification via the aldose reductase (ALR2) pathway, possibly as a function of substrate concentration in consequence to hemi-thioacetal formation [38]. However, GSH at concentrations up to 2 mM enhanced Glo1 activity *in vitro* [39].

Thiol-exchange mechanisms have been discussed for glutathionylation, but, unless the protein has an unusual redox potential, they would require high concentration of GSSG, which is not likely to occur *in vivo* [40]. Formation of reactive sulfhydryl intermediates of protein-SH and GSH most likely precedes protein glutathionylation *in vivo*, such as *S*-nitrosyl (–SNO) and other (sulfenic acid –SOH, thiyl radical, thiosulfinate –S(O)SR, or sulfenyl-amide, cyclic-S–N–CO–). Increase in the GSH/GSSG ratio or enzymatic reactions involving protein disulfide isomerase, glutaredoxin 1 (GRx1) or thioredoxin, were suggested to restore the protein sulfhydryls to their reduced state. Currently, proposed candidates to catalyze *S*-glutathionylation *in vivo* include glutathione-*S*-transferase pi and GRx1. [28]

Nitrosylation is one way suggested to activate protein thiols prior to glutathionylation. Human Glo1 has been shown earlier to be responsive to nitrosylation by *S*-nitroso-gluthathione (GSNO): the presence of a faster migrating isoform of Glo1 on non-denaturing gels was observed after treatment of L929 cells with increasing concentration of GSNO [12]. The authors concluded that the NO-mediated modification of enzymatic activity of Glo1 is possibly the consequence of structural changes that are induced by this modification. However, considering the increased net charge of the detected isoform, glutathionylation was denied, which would in fact have lead to a decrease of net charge. In contrast, our data clearly indicate that glutathionylation does occur at Cys^139^; other modifications such as the state of oxidation of the vicinal Cys^19^ and Cys^20^ could contribute to the net charge as well.

### Glutathionylation vs. disulfide bridge on Cys^139^

Beside glutathionylation on Cys^139^, we found this same residue to form an optional disulfide bridge with Cys^61^. Since Cys^61^ was found so far in human only, this disulfide would be unique to human. Consequently, Cys^139^ might be involved in at least two alternatively occurring posttranslational modifications of Glo1 in human, underpinning the crucial role this residue obviously plays in regulation of the enzyme. We found evidence for the presence of at least two modifications of Cys^139^ in native human Glo1 beside the free thiol, i.e. the mixed disulfide with GSH, and bound in a disulfide bridge with Cys^61^.

**Figure 5:**
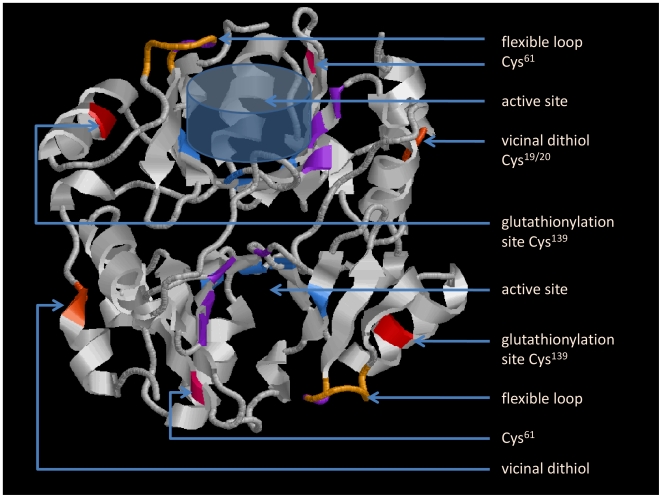
3D structure of a Glo1 dimer according to [24]. Colored residues show the position of Zn-ligands (blue) and GSH-ligands (magenta). The transparent blue cone placed in the upper monomer mimics the general position of a ligand in the barrel containing the active site. The flexible loop that closes upon ligation over the barrel is colored in gold, the cysteine residues in shades of red. We suggest that covalent binding of GSH to Cys^139^ brings about a conformational change to the flexible loop that might subsequently close the barrel.

In a study by Lan [41], the presence of a flexible loop near the active site of Glo1 was proposed which was specified later on by Creighton and coworkers [24] (Figure 5). The hydrogen bonding pattern of this loop (residues 153–160) in the open and closed conformations suggested that the loop is open in the absence of bound ligand, which enables the substrate (or inhibitor) to enter the active site. After binding of enediolate analogues, the loop closes above the active site [24]. Though for interaction with the inner sphere ligands of the essential metal Zn (Figure 5), an *S* substituent on glutathione is necessary, an *S* substituent was not required for ligation of GSH alone [42]. Thus, if GSH simply ligates with Glo1, the flexible loop closes and prevents other substrates from entering. GSH as enzyme ligand was found to interact mainly with the guanidino group of Arg^38^ and the amide group of Asn^104^ forming hydrogen bonds with both, the carboxylate and the amino group of the γ-glutamate of GSH [9], but not Cys^139^.

According to [24], Cys^139^ should be rather located near the flexible loop (Figure 5). Covalent binding of GSH to Cys^139^ as found in our experiments might also induce an inactive conformation by moving the flexible loop over the active site of the enzyme and thus bring about suppression of enzyme activity by preventing the hemithioacetal substrates from entering, such as MGO-GSH which was tested in our experiments. A disulfide between Cys^61/139^ (Figure 5) could even close the barrel completely.

**Figure 6:**
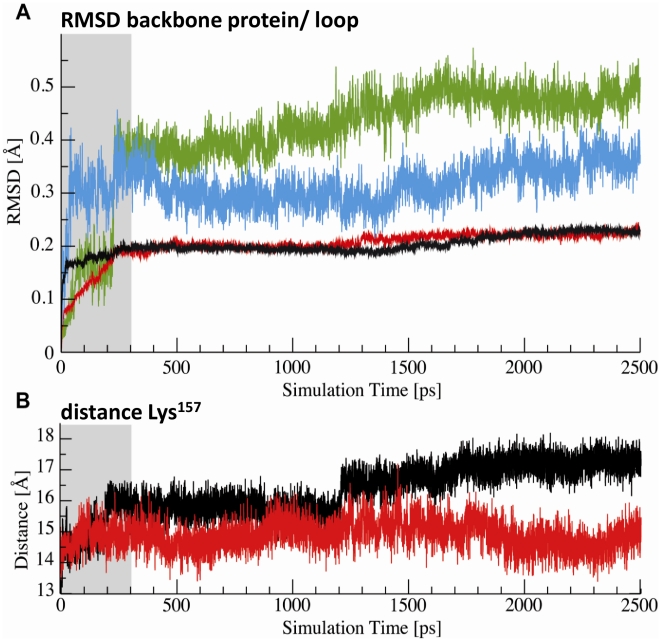
Molecular simulations of Glo1 dimer. The grey area denotes the heating phase (cf. text). (A) Root mean square distances (rmsd) of the backbone atoms of the loop (153–160) in the glutathionylated protein (green) and the unmodified protein (blue). The rmsd of the rest of the protein remains stable. (B) Distance between the Zn^2+^ ion and the Cα atom of Lys^157^. The distance in the glutathionylated protein (red) is smaller than that in the unmodified protein (black) over the entire simulation period.

A simulation of molecular dynamics was carried out to test this hypothesis (Figure 6). As a result of the calculations it turned out that the geometry of the backbone of the protein itself did not significantly change upon of GSH binding to Cys^139^ with exception of the loop involving residues 153–160. Root mean square deviation of the loop backbone itself indicated an obvious structural change when comparing the experimentally determined configuration [24] with the computed glutathionylated protein (Figure 6A): the distance between the active Zn^2+^ ion and the C_α_-atom of Lys^157^ became smaller suggesting a movement of the flexible loop directed towards the active site of the protein upon glutathionylation (Figure 6B) which would be in full agreement with our assumptions.

De Hemptinne et al. [12] investigated the role of all four cysteines in NO-mediated modification of Glo1 and found that replacement of Cys^61^ by exchange with alanine did not prevent from NO-modification after GSNO treatment, while the Cys^139^ > Ala mutant, was impaired for NO-mediated modification. Notably, we found Cys^139^ as the target for glutathionylation. It was suggested that GSNO treatment-mediated change in enzyme activity was brought about by a conformational change in agreement with our hypothesis concerning the flexible loop.

Glutathionylation was found earlier to precede reversible formation of an intramolecular disulfide bridge in many proteins [43]. It has been shown that formation of an intramolecular disulfide after glutathionylation in low molecular weight protein tyrosine phosphatase (LMW-PTP) was involved in rescuing enzyme activity via restoring the reduced state after oxidation of the cysteine located in the catalytic pocket [44]. An intriguing thought would be that formation of a Glo1 intramolecular disulfide between Cys^61^ and Cys^139^ could exhibit a similar function, e.g. to reduce bound GS(H) and subsequently remove it in a first step to restore enzyme activity.

### The vicinal disulfide Cys^19/20^

Vicinal cysteines are rather rare in proteins, the more if found near the *N*-terminus distant from the active site of the enzyme. The redox state of these cysteins is likely having other functions than mere protection from degradation [45], [46]. An interaction with the enzyme's environment in a redoxstate-relevant situation becomes even more indicative considering that the Glo1 *N*-terminus is exposed to the surface in the tertiary structure of the enzyme (Figure 5).

Vicinal disulfides are generally suggested to exert different functions, such as receptor and co-factor binding, or a role in oxidative folding. Oxidized vicinal disulfide bridges impact protein folding; the peptide is forced in a distorted trans-conformation. Due to the conserved tertiary structure of the vicinal disulfide turn and the dramatic change that can be expected upon reduction of it, this structural element was suggested to act as a redox-activated conformational switch [45]. Disruption of such thiols was shown to inactivate the redox-sensitive protein phosphatase [47] as well as squalene monooxygenase [48] and affected the function of many other proteins [45], [49], [50].

De Hemptinne et al. [12] found the cysteine residues Cys^19/20/139^ together involved in responsiveness of Glo1 to GSNO-treatment in human red blood cells. NO-mediated modification was maintained for Cys^19^ and Cys^20^ single mutants, impaired for the double mutant Cys^19/20^ and completely absent in triple mutants of Cys^19/20/139^. It was suggested that Cys^139^ interacts with either Cys^19^ or Cys^20^ for this modification of the protein.

The vicinal thiol-specific reagent phenylarsine oxide (PAO) significantly enhanced glutathionylation in oxidatively stressed cells [51]. Again, in a reducing environment such as the cytoplasm, disulfide bonds are expected to serve a certain function; an intriguing thought would be that Cys^19^ and Cys^20^ could play a role in mediating the proposed glutathionylation or, respectively, dethiolation of Cys^139^ itself, which could eventually explain the differential interaction of these three cysteine residues in NO-mediated modification of Glo1 [12] and be consistent with our results, where enzyme activity was not suppressed with PAO only.

### Conclusions

The active role of GSH in redox regulation of different processes was recently addressed in several studies. Thus, our findings of a posttranslational modification of Glo1 by GSH could be important for the many diseases, in which altered Glo1 activity was observed and, consequently, obviously might need to be regulated in response to the redox state of tissues beyond protein expression, such as tumor cell proliferation [6], diabetes [52], congenital nonspherocytic hemolytic anemia [53], and numerous others.

In particular, reversible inhibition of Glo1 by glutathionylation is certainly expected to impact detoxification of cellular MGO as depleted GSH and elevated MGO concentration were characterized as key events in glyoxalase inhibition [54]. High concentrations of MGO are toxic to cells, for instance by secondary depletion of ATP, modulation of mitochondrial membrane potential, induction of apoptosis, and ROS-production [55]. Recent results showed that MGO also has regulatory functions on proteins such as activation of transcription factors [55], modulation of enzyme activities [56], and inhibition of NF-κB p65 binding to DNA [57]. Under anaerobic conditions, glycolysis is triggered in the cell and MGO production is enhanced, so that the cell could use redox-regulation of Glo1 to respond immediately to enhance detoxification with MGO without detour of transcription and protein synthesis.

Very clearly, we showed that oxidation of Cys^139^ by means of glutathionylation caused by GSSG has impact on enzyme activity. Three of the five identified posttranslational modifications are in fact disulfides, strongly suggesting that Glo1 is indeed regulated in response to the redox state of the cell.

## Materials and Methods

### Materials and chemicals

Ammonium acetate (puriss. p.a., ACS reagent, ≥98.0%), iodoacetamide (purum ≥98.0%), formic acid (puriss. p.a., for mass spectroscopy), trifluoroacetic acid (TFA, for UV-spectroscopy), and ammonium bicarbonate (NH_4_HCO_3_, ≥99.5%) were obtained from Fluka Chemie GmbH (Buchs, Switzerland). Porcine trypsin (Sequencing Grade Modified Trypsin) was purchased from Promega (Madison, USA). Acetonitrile (LC-MS grade) was obtained from Biosolve B.V. (Valkenswaard, Netherlands). Pipette tips for sample preparation (C18, ZipTip™) were obtained from Millipore (Billerica, USA.). All aqueous solutions were prepared with deionized water having a resistance of at least 18 MΩ x cm (Elga–Labwater, Celle, Germany). α-Cyano-4-hydroxycinnamic acid (CHCA) for MALDI analyses were purchased from Bruker Daltonics GmbH (Bremen, Germany). Rotiphorese Gel 30 (acryl amide and N,N'-Methylene bisacryl amide 37,5:1), reduced L-glutathione (GSH), soybean trypsin inhibitor, and carboanhydrase were purchased from Roth GmbH (Karlsruhe, Germany). Oxidized glutathione (GSSG) was obtained from Boehringer (Mannheim, Germany). Anti-GSH mouse monoclonal antibody (anti-GSH mab) was obtained from Virogen (Watertown, USA) and anti-Glo1 monoclonal antibody (Clone 4C10, anti-Glo1 mab) from BioMac GmbH (Leipzig, Germany). Horseradish (HRP)-labeled goat anti-mouse Immunoglobulin (Ig) was delivered by Dako (Glostrup, Denmark). Dithiothreitol (DTT), β-mercaptoethanol (β-ME), 3,3′-diaminobenzidine (DAB), Phenylarsine oxide (PAO), *S*-hexylglutathione sepharose, *S*-hexylglutathione, methylglyoxal (MGO), Phenylmethylsulfonylfluoride (PMSF), Tween 20, and bovine serum albumin (BSA) were purchased from Sigma (Taufkirchen, Germany). Coomassie Brilliant Blue R250 and Ponceau S were ordered from Serva (Heidelberg, Germany). All other chemicals were of analytic reagent grade.

For visualization of the 3D structure of Glo1 dimer according to [24], Open Source RasWin Molecular Graphics, windows version 2.7.5., was used. The protein data bank file 1QIN.pdb used for Figure 5 was downloaded on 04/09/2008 from http://www.pdb.org/.

The same file was used for calculations of molecular dynamics with the modeling package MOE 2008.10 (Chemical Computing Group, Inc. Montreal, Canada). After removal of the crystallographic water molecules, hydrogen atoms were added and Amber99 charges assigned. The simulation of glutathionylation on Cys^139^ was performed using the molecular editor module of MOE. For MD-simulation, the system was slowly heated up to 300 K during 300 ps. The system was simulated *in vacuo* for 2300 ps with a time step of 2 fs according to the MOE 2008.10 manual. A snapshot was saved every 0.1 ps. Before analysis, the backbone atoms of each snapshot were aligned to the starting structure.

### Purification of Glo1

Glo1 was purified from human erythrocytes under conditions preventing from oxidation according to Mannervik et al. [58] yielding 90% enzyme purity (SDS-PAGE). Specific enzyme activity of various Glo1 preparations ranged between 450 and 700 U/mg. Fresh human erythrocytes were obtained from the Blood Transfusion Centre of the Medical Faculty of the University of Leipzig and were handled according to regulations of the local authorities, including ethics approval of our study by the Ethics Committee of the University of Leipzig (Vote No. 146-2006) and written informed consent from all participants.

### Mass spectrometry of the intact monomer by ESI-FTICR-MS

Purified Glo1 dissolved in 20 mM ammonium acetate/acetonitrile 2:1 with 0.1% or 1% formic acid (v/v), respectively, was subjected to flow injection analysis by ESI-FTICR-MS in positive mode. For all ESI analyses, a BioApex II ESI-FTICR-MS instrument (Bruker Daltonics, Bremen, Germany) with XMass software was used, equipped with a 7.4 Tesla magnet and an electrospray ion source (Agilent, Waldbronn, Germany). For treatment with β-ME, 0.1 mg freeze-dried Glo1 was dissolved in 20 mM ammonium acetate, pH 7.0, and incubated at room temperature in 25 mM β-ME prior to measurement.

### Tryptic digest of Glo1, alkylation and reduction of disulfide bonds

Freeze-dried Glo1 (0.1 mg) was dissolved in 3 mM ammonium bicarbonate (ABC), pH 8.5, and was divided into two equal aliquots. To one aliquot, 1 M DTT was added and the reaction mixture was subsequently incubated at 95°C for 5 min. After centrifugation, the sample was incubated with iodoacetamide solution (27.5% w/v in water). The sample was incubated at room temperature and conditioned with 150 mM ABC. The second aliquot was incubated with 3 mM ABC only. Both aliquots were processed further identically by incubation with trypsin solution (20 ng/µl in 3 mM ABC) at 37°C overnight. After centrifugation, formic acid was added and the samples were dried in a speed vac. Samples were dissolved again in 0.1% formic acid and purified on C18 ZipTip. Peptides were eluted with 0.5% formic acid in 80% acetonitrile and subjected to analysis by mass spectrometry. The non-reduced sample was then reduced following the procedure using iodoacetamide and DTT described above and analyzed again. In a second experiment, alkylation with iodoacetamide only was carried out to confirm obtained results for the non-reduced preparation.

### Analysis of tryptic digests by mass spectrometry

The tryptic digests were analyzed by MALDI-TOF/TOF-MS/MS in positive ion mode, (4700 proteomics Analyzer Applied Biosystems, Applera Deutschland GmbH, Darmstadt, Germany) with α-cyano-4-hydroxycinnamic acid (CHCA) as matrix. ESI-FTICR-MS analyses of the digests were carried out as described above for the intact monomer.

Alternatively, an HPLC 1100 series (Agilent Technologies GmbH, Waldbronn, Germany) equipped with a Zorbax 300 SB-C18 column (column dimensions 150 mm×75 µm, particle size 3.5 µm; Agilent Technologies GmbH, Waldbronn, Germany), was coupled on-line to the nanoESI-source (Proxeon, Odense, Denmark) of a Qq-TOF-type mass spectrometer (QSTAR Pulsar I, Applied Biosystems, Applera Deutschland GmbH, Darmstadt, Germany). 1 pmol of the non-reduced tryptic digest was dissolved in 3% acetonitrile and injected. Eluents were 3% acetonitrile containing 0.1% formic acid (eluent A) and 0.1% formic acid in acetonitrile (eluent B). The flow rate was 0.25 µl/min. The sample was loaded isocratically with 3% eluent B for 10 min. Elution was performed by a 60-min gradient (1–45% eluent B in 30 min, 45–75% eluent B in 10 min, 75–90% eluent B in 10 min, 90–3% eluent B in 10 min). Ion spray voltage was set to 2200 V. The two most intense, double to quintuple charged signals detected in a TOF experiment were selected to perform automated MS/MS analysis of each parent ion for 1 s each in the m/z range from 70 to 1700. The spectra were interpreted with Analyst QS software (Applied Biosystems, Applera Deutschland GmbH, Darmstadt, Germany) and MASCOT database (Matrix Science Ltd., London, UK).

### Glyoxalase assays

Glo1 activity was measured according to Mannervik et al. [58].

To study the effect of glutathione depletion, enzyme activity of 100 µl purified Glo1 (5 units) with a specific activity of 570 U/mg was measured after incubation without and with 5 mM and 20 mM β-ME followed by dialysis (4°C, 2 h) against 50 mM sodium phosphate buffer, pH 7.0.

The effect of GSSG on enzyme activity was assessed by activity measurement following incubation of de-glutathionylated enzyme with increasing concentration of GSSG. To this end, after treatment with 20 mM β-ME, Glo1 was incubated with 5 mM and 20 mM GSSG at room temperature.

Aliquots (20 µg) of native, β-ME- and GSSG-treated enzyme (each at a concentration of 20 mM), were subjected to SDS-PAGE under non-reducing conditions followed by Western blotting. The membrane was stained for protein with Ponceau S and subsequently probed with the anti-GSH Ab. The enzyme activity of all samples was determined.

### Kinetic analyses

Enzyme activity (ΔE/min) was determined upon incubation of non-treated and β-ME-treated enzyme with increasing equimolar concentrations of MGO/GSH. Reduction of Glo1 (5 units) was carried out by incubation with 20 mM β-ME (30 min, 22°C), followed by dialysis against 50 mM sodium phosphate buffer, pH 7.0 (2 h at 4°C). Kinetic data were fitted to the Michaelis-Menten equation using the Marquardt-Levenberg method with simple weighing. A goodness-of-fit criterion (Akaike's information criterion, AIC) was used for falsification of sigmoid enzyme kinetics. The quality of the fit was characterized by 95% confidence intervals of the estimated parameters. The analysis was performed using the Enzyme Kinetics Module of SigmaPlot (Systat Inc., San Jose, USA).

### Western blot of Glo1

Proteins were separated by SDS-PAGE in pore gradient gels (4% to 20% T) under non-reducing conditions and blotted to cellulose nitrate membranes (Whatman Schleicher & Schuell, Dassel, Germany) stained with Ponceau S. After blocking with 3% defatted milk over night, the membrane was probed with mouse anti-GSH mAB (1 µg/ml), and with mouse anti-Glo1 mAB (1 µg/ml) in 50 mM sodium phosphate, 150 mM sodium chloride, pH 7.0 (PBS), containing 3% BSA and 0.1% Tween 20 (PBS-T-BSA) for 1.5 h. After washing with PBS containing 0.1% Tween 20 (PBS-T), the membrane was incubated with horse radish (HRP)-labelled goat anti-mouse Ig (1∶1000). Color was developed with DAB/H_2_O_2_. Proteins separated by SDS were stained with Coomassie Brilliant Blue R250. Soybean trypsin inhibitor (20 kDa) and carboanhydrase (29 kDa) were used as molecular mass markers.

### Coupling of glutathione to bovine serum albumin

BSA (5 mg/ml) was incubated with the cross-linking reagent N-succinimidyl-3-(2-pyridyldithio) propionate (SPDP) (Pierce, Bonn, Germany) (0.34 mg in 100 µl ethanol) in 50 mM sodium phosphate buffer, pH 7.0 at 20°C for 30 min. The sample was dialyzed against incubation buffer and reacted with 5 mg GSH. The reaction mixture was dialyzed against phosphate buffer overnight. The formation of pyridine-2-thione was assessed at 343 nm to calculate bound GSH. GSH/BSA ratio of the final product was 2.5 mol/mol.

In this report, all amino acids were numbered according to Ref_prot NP_006699.
